# Capital Punishment

**Published:** 1912-09

**Authors:** 


### EDITORIAL DEPARTMENT.

DR. F. E. DANIEL, Editor DR. R. H. L. BIBB, Associate Editor

DEPARTMENTS { EUGENICS: Drs. M. Duggan and T. Y. Hull.

WOMAN'S DEPARTMENT: Mrs. F. E. Daniel.

### Capital Punishment.

THE SLAUGHTER PEN AT SING SING.

Of all the absurd, senseless, useless statutes on the law books, that of the death penalty for crime is the most so! The stock argument in defense of it is that "it vindicates the majesty of the law"; "it deters evil-doers." Just how much it deters them is evidenced by the fact that crime,—"capital offenses,"—is on the increase, and recently the social fabric of not only New York City but of the whole country was shaken by the cold-blooded murder of a man named Rosenthal, by a gang of hired assassins in the presence of a large number of spectators, in the glare of hundreds of electric lights in the very heart of the city. But a greater shock followed when the papers announced the wholesale electrocution at Sing Sing, when seven murderers were killed in the horrible electric chair, in two hours. It is sickening to contemplate! It is disgusting. It resembles the horrors of the Swift and Armour abattoirs at Chicago! It will lead to the abolition of the death penalty for crime, because public sentiment revolts at it. Mrs. Champ Clark has started a movement in that direction, and it will sweep the country like a prairie fire. I wish it success. The death penalty should be abolished as worse than useless. Six of the seven murderers slaughtered at Sing Sing were Italians. Here our immigration laws are at fault; that class should never have been permitted to enter, or being here should have been deported.

It is perhaps an easy matter to show the absurdity of the death penalty and its failure of its ostensible ends,—the State essays to purify the morals of society by perpetrating a shocking crime. The law—human and divine—says "thou shalt not kill,"—and the State gives us an object lesson in killing,—in cold blood.

But, what are you going to do with the man-killers? The problem has not been solved; many expedients have been suggested, none of which seems to be satisfactory. To put them to work under restraint—for the benefit of that society whose privileges have been forfeited—seems the most reasonable, but there the cruelties and tortures permitted by brutal guards renders penitentiary life worse than electric chair death. I give it up.

I could make a strong plea for the killer on the score that probably he is not responsible—since perhaps he has inherited the propensity from ancestors; it may be alcoholics! But it seems useless to argue; the killing goes on, and, doubtless, always will go on, but maybe, after awhile, the State will realize the inutility of *retaliation in kind* and stop the "legal" murders; it may realize some day that the law, in a measure, at least, is responsible. Whose fault is it that a boy becomes a criminal? The State should see that the young are qualified by education, timely aid and employment to become citizens, and not allowed to become criminals—as they are sure to do, idle and ignorant. And, where the State metes out so-called "justice" by punishing (killing) a man for crime committed under the influence of liquor, it is worse than a farce to call it justice; it is the rankest kind of injustice. The State licenses the sale of liquor, solely for the revenue derived thereby. It thus aids and abets the saloon keeper to tempt the young, the weak, the reckless and the unwary to put that into their stomachs which robs them of reason, for the time being, and deprives them of the power to resist an evil impulse.

It has been said that the way to get rid of a bad law is to enforce it. The killing law has been enforced at Sing Sing *ad nauseam*. Let us hope that a reaction will take place, and the people demand the repeal of the cruel, savage, brutal and useless code. It is out of date in an enlightened age and amongst an enlightened and "progressive" people.

